# Corrigendum to: Genetic Associations in Four Decades of Multienvironment Trials Reveal Agronomic Trait Evolution in Common Bean

**DOI:** 10.1093/genetics/iyab158

**Published:** 2021-10-15

*Genetics*, 2020, https://doi.org/10.1534/genetics.120.303038

In the originally published version of this manuscript, there are errors in the presentation of error bars in Figure 3D, 3E, and 3F. The figure legend incorrectly reports the error bars as standard errors, but they are in fact standard deviations.

The legend for Figure 3 should instead read as follows:

“Patterns of phenotypic effects of genetic associations for 22 phenotypes from the CDBN, determined using multivariate adaptive shrinkage. (A) SNPs with significant effects on 1 or more of the 22 phenotypes in the CDBN. (B) Manhattan plot of the Bayes factor (log_10_) comparing the model likelihood that the SNP has significant effects to the likelihood that it has no significant effects. Bayes factors of > 10^2^ are considered decisive evidence in favor of the alternate model. Point color represents the number of phenotypes for which the SNP has a local false-sign rate < 0.05. Squares represent even chromosomes, while circles represent odd chromosomes. The top associations for three regions of the genome are highlighted. (C) Correlation in the sign and magnitude of significant effects in all pairwise comparisons of the 22 CDBN phenotypes. Circle size and color indicate the fraction of all significant SNPs that have the same effect sign and similar effect magnitude. (D–F) Effect estimates and standard deviations for 22 phenotypes for the top associations from three regions of the genome, (D) *P. vulgaris* chromosome 1 (Pv01) at 15.4 Mb, (E) Pv01 at 42.2 Mb, and (F) Pv07 at 14.5 Mb. Genomic locations are based on the *P. vulgaris* v2.1 genome annotation. Standard deviation bars are colored by the six groups present in (C). CBB, common bacterial blight; CDBN, Cooperative Dry Bean Nursery; CTV, curly top virus.”

These details have been corrected only in this corrigendum to preserve the published version of record.

